# ASF1B promotes gastric cancer progression by modulating H2AC20 and activating PI3K/AKT and ERK1/2 pathways

**DOI:** 10.3389/fphar.2025.1533257

**Published:** 2025-02-18

**Authors:** Mengyuan Zhao, Junchang Zhang, Yanjun He, Chongge You

**Affiliations:** ^1^ Laboratory Medicine Center, The Second Hospital and Clinical Medical School, Lanhzou University, Lanzhou, China; ^2^ Department of Anesthesiology, Shandong Provincial Hospital Affiliated to Shandong First Medical University, Jinan, Shandong, China

**Keywords:** gastric cancer, AFS1B, H2AC20, PI3K/AKT, ERK1/2

## Abstract

**Background:**

Gastric cancer (GC) ranks among the most prevalent malignant neoplasms globally and is associated with a significant mortality rate. Despite the availability of various therapeutic interventions for GC, the overall prognosis for this disease remains unfavorable. This can be attributed to several factors, including delayed diagnosis and the inherent heterogeneity of the tumors. With the continuous enrichment of treatment methods, GC has entered an era of comprehensive treatment oriented toward precision and standardization.

**Methods:**

Through the application of bioinformatics and assessments of tissue microarrays, this study has selected the histone chaperone Anti-Silencing Function 1B (ASF1B) for detailed analysis, including clinical specimens. We then constructed ASF1B knockout and overexpression cell lines, and conducted biological function tests on this basis, validated at mouse and organoid levels. Additionally, human immunereconstitution was performed in NOD-PrkdcscidIl2rgem1/Smoc (NSG) mice, followed by flow cytometry analysis of mouse blood. Mechanically, protein-protein interaction analyses were conducted utilizing Immunoprecipitation-Mass Spectrometry (IP-MS) and Tandem mass tagging (TMT) methodologies to identify protein clusters.

**Results:**

The analysis demonstrated that ASF1B is significantly upregulated in GC tissues and correlates with unfavorable prognostic outcomes. Biological function tests provided that ASF1B contributes to tumor cell proliferation, colony formation, invasion and migration, and plays an important role in the progression of GC *in vivo*. These findings were validated at both the mouse and organoid levels. Additionally, we observed that ASF1B is involved in the tumor microenvironment, where ASF1B knockdown increases CD8^+^ T cell infiltration, indicating a negative correlation with immune activation. Mechanically, our investigation revealed that ASF1B emerged as a promoter of GC progression by downregulating H2A clustered histone 20 (H2AC20), thereby influencing the activation of the phosphoinositide 3-kinase (PI3K)/protein kinase B (AKT) and extracellular regulated protein kinases (ERK)1/2 signaling pathways.

**Conclusion:**

ASF1B, recognized as an oncogene, contributes to the initiation and progression of tumors, positioning it as a prospective target for therapeutic intervention in GC.

## 1 Introduction

Gastric cancer ranks among the predominant malignancies globally, characterized by a significant mortality rate. Recent cancer statistics indicate that GC stands as the fifth most frequently diagnosed cancer and the fourth primary contributor to cancer-related fatalities internationally (Sung et al., 2021). Even with the observed decrease in incidence rates, projections indicate that the worldwide impact of this cancer is anticipated to rise by 62% by the year 2040 (Thrift et al., 2023). Surgery and endoscopic resection are the primary treatments, particularly for early-stage diseases. For patients with advanced disease, chemotherapy and targeted therapy can improve survival and quality of life. However, the benefits of these strategies are limited, with a mortality rate of up to 75% in most parts of the world (Thrift et al., 2023). Therefore, elucidating the mechanisms underlying GC progression and identifying effective therapeutic strategies are crucial to increasing the chances of a cure for GC patients.

Alterations in host genetic factors play a critical role in the molecular underpinnings of cancer. Aberrant gene expression, which encompasses gene silencing and overexpression, correlates with DNA methylation variations and histone post-translational modifications’ irregularities. Research has shown that the modulation of chromatin regulators—comprising histone variant proteins, histone chaperone proteins, histone modifying enzymes, effector proteins, and chromatin remodeling proteins—relates significantly to cancer initiation and advancement (Gurard-Levin and Almouzni, 2014; Gurard-Levin et al., 2014). The H3-H4 chaperone protein, anti-silencing function 1 (ASF1), represents a crucial histone chaperone involved in the chromatin-mediated regulation of cellular processes such as DNA replication, DNA damage repair, and transcription. ASF1 has two paralogs, namely, ASF1A and ASF1B. ASF1A is predominantly responsible for facilitating DNA repair and promoting cellular senescence, whereas ASF1B is more selectively associated with cell proliferation (Abascal et al., 2013; Paul et al., 2016).

In previous studies, ASF1B has been found to promote the growth of myeloma (Misiewicz-Krzeminska et al., 2013; Wang et al., 2020), glioma (Zhang and Liu, 2023), and cancers in breast (Corpet et al., 2011), kidney (Chen et al., 2020), prostate (Carrion et al., 2020), bladder (Zhang et al., 2024) cervical (Xu et al., 2019; Liu et al., 2020), lung (Zhang et al., 2021; Song et al., 2024), liver (Zhang et al., 2022a), and stomach (Zhao et al., 2024; Zhang et al., 2023) as an oncogene. Its expression is significantly associated with survival prognosis in most solid tumors. Recent evidence (Song et al., 2024; Hu et al., 2021a) also suggests that ASF1B may serve as a prognostic biomarker related to immunotherapy in several cancers. Nevertheless, the precise mechanisms and regulatory networks through which ASF1B influences GC development necessitate additional research. This study aimed to elucidate the role of ASF1B in GC development and identify H2AC20 as a downstream effector of ASF1B. Our findings demonstrate that ASF1B knockdown significantly inhibits GC cell proliferation both *in vitro* and *in vivo*. Furthermore, the results suggest that ASF1B regulates GC cell growth and metastasis through modulation of H2AC20 expression.

## 2 Materials and methods

### 2.1 ASF1B expression analysis using bioinformatics data

The mRNA expression data, along with associated clinical details for GC patients, were obtained from The Cancer Genome Atlas (TCGA) and Genotype-Tissue Expression (GTEx) databases. The pancancer expression profile of ASF1B was illustrated using the TIMER platform (cistrome.shinyapps.io/timer). To delve deeper into ASF1B expression specifically in GC, RNA-seq gene expression data from the tissues of 407 GC patients were extracted from the TCGA database (https://xenabrowser.net/datapages) for analysis. Furthermore, the top 200 genes linked to patient survival were obtained from the GEPIA2 database.

### 2.2 Immune infiltration analysis

The correlation between 24 immune cell subsets in samples and controls was assessed using Pearson correlation analysis and visualized with the ggplot2 R package.

### 2.3 GO and KEGG pathway analysis

Genes with a LogFC greater than 1 relative to ASF1B expression were defined as ASF1B-related genes. The c5.go.v7.2.symbols.gmt and c2.cp.kegg.v7.2.symbols.gmt datasets in the Molecular siqnature Database (MsigDB) were imported into the GSEA 41.0 software for GO analysis and KEGG pathway enrichment analysis of CLDN-7. P < 0.05 was considered statistically significant.

### 2.4 Gene set enrichment analysis

Gene set enrichment analysis (GSEA) was conducted using GSEA software (version 4.0.3). Samples from the TCGA database were divided into two groups based on ASF1B expression levels. The c5.go.v7.2.symbols.gmt and c2.cp.kegg.v7.2.symbols.gmt data sets from the Molecular Signature Database (MsigDB) were imported into GSEA 4.1.0 software for GO and KEGG pathway enrichment analysis. A p-value <0.05 was considered statistically significant.

### 2.5 Cell culture

Human gastric cancer cell lines AGS, SNU216, HGC-27, and MKN-45 were sourced from the Chinese Academy of Medical Sciences, while HEK-293T cells were obtained from the American Type Culture Collection (ATCC). These cell lines were cultured in Dulbecco’s Modified Eagle Medium (DMEM),which was supplemented with 10% fetal bovine serum, 100 U/mL penicillin, and 100 mg/mL streptomycin. All cell lines underwent authentication through STR profiling.

### 2.6 Lentivirus construction

Lipofectamine 2000 (Invitrogen), pLenti-CRISPR-V2, pSPAX2, and pMD2G plasmids were transiently transfected into HEK293T cells. After 60 h, the supernatant was collected and centrifuged at 12,000 rpm. The lentivirus suspensions were filtered using 0.22 μm filters and used to infect MKN45 and HGC27 cells at a concentration of 1 × 10^∧^8/mL for 48 h. Puromycin was then used to select stable ASF1B knockout GC cell lines, and the knockout efficiency was determined by Western blot (WB). The guide RNA (gRNA) sequences used for CRISPR/Cas9-mediated ASF1B knockout are listed. For ASF1B and H2AC20 overexpression, lentiviral constructs and controls were purchased from GeneChem (Shanghai, China), and cells were transfected according to the manufacturer’s guidelines.

**Table:** 

FWI	TGT​GGA​AAG​GAC​GAA​ACA​CCG​GAA​GCT​GAT​CTC​GAA​CCG​GAG​TTT​TAG​AGC​TAG​AAA​TAG​CA
RVI	TGC​TAT​TTC​TAG​CTC​TAA​AAC​TCC​GGT​TCG​AGA​TCA​GCT​TCC​GGT​GTT​TCG​TCC​TTT​CCA​CA
FWII	TGT​GGA​AAG​GAC​GAA​ACA​CCG​CAT​CCC​AGA​GAC​TGA​TGC​CGG​TTT​TAG​AGC​TAG​AAA​TAG​CA
RVII	TGC​TAT​TTC​TAG​CTC​TAA​AAC​CGG​CAT​CAG​TCT​CTG​GGA​TGC​GGT​GTT​TCG​TCC​TTT​CCA​CA

### 2.7 Human gastric cancer clinical specimens

All specimens were acquired from patients under the protocols approved by the Human Research Ethics Committee of Lanzhou University Second Hospital (2020A 052). A total of 16 groups of gastric cancer and adjacent normal tissues were used for microarray analysis to obtain differentially expressed genes. In total, 664 proteins were identified as being significantly overexpressed in GC tissues compared to adjacent normal tissues. Fresh tumor samples along with adjacent normal tissues were collected from patients with GC (n = 4). Total RNA was extracted, and its quality was assessed using a NanoDrop 2000 and an Agilent Bioanalyzer 2100. mRNA microarray analysis was conducted utilizing the Affymetrix GeneChip, with fluorescence signals captured by the Affymetrix GeneChip Scanner 3000.

### 2.8 Cell viability analysis

Cell viability was assessed utilizing the MTT assay. Approximately 2 × 10^∧^3 cells were plated into a 96-well plate and incubated for intervals of 24, 48, 96, 120, and 144 h. Following this, 20 μL of MTT reagent was introduced to each well and incubated at 37°C for 2 h. Absorbance readings were taken at 490 nm with a microplate reader. All experiments were conducted in duplicate, comprising three replicate wells for each group.

### 2.9 Colony formation analysis

Approximately 1,000 GC cells were uniformly inoculated in 35 mm Petri dishes. Following a cultivation period of about 2 weeks, the wells were rinsed with phosphate-buffered saline (PBS), fixed using 4% paraformaldehyde, and stained with 0.05% crystal violet. The resulting images were processed with ImageJ software and analyzed using GraphPad Prism 8.0.

### 2.10 Transwell invasion analysis

Approximately 200 μL of a GC cell suspension (10^∧^5 cells/mL) was meticulously introduced into the Matrigel-coated upper chamber of a Transwell. Serum-free medium was added to the upper chamber, while the lower chamber received medium enriched with 10% fetal bovine serum (FBS). After a 48-h incubation period, the cells were rinsed with PBS, gently swabbed, fixed with 4% paraformaldehyde, and stained overnight with 0.05% crystal violet. The stained cells at the bottom of the invasion chamber were then imaged using a microscope and analyzed utilizing GraphPad Prism 8.0 software in conjunction with ImageJ.

### 2.11 Cell scratch migration analysis

Approximately 3 × 10^∧^5 GC cells were plated in a 6-well plate. The following day, a 200 μL pipette tip was employed to create parallel linear scratches on the cell monolayer. Cells were maintained in serum-free medium and imaged using a microscope. After a 48-h incubation period, images of the same field of view were captured, and the scratch closure rate was quantified utilizing GraphPad Prism 8.0 software in conjunction with ImageJ.

### 2.12 Flow cytometry analysis

After an initial period of starvation, the cells were cultured for 24 h in a complete medium that was enriched with 10% FBS. Following this, the cells were treated with trypsin for digestion, subjected to centrifugation, and then resuspended in 70% pre-cooled ethanol. They were subsequently incubated at 4°C overnight. The next day, the cells underwent another round of centrifugation and were rinsed with PBS to eliminate any residual ethanol. They were then resuspended in 300 μL of propidium iodide (PI) master mix and incubated in a dark and humid environment for 30 min. Cell cycle progression was assessed via flow cytometry, and the resultant data were analyzed using ModFit LT software.

### 2.13 Apoptosis analysis

The Annexin V-FITC Apoptosis Detection Kit (Beyotime Institute of Biotechnology) was used to detect cell apoptosis. Cells were collected in 5 mL tubes, centrifuged at 1,000 × g for 5 min at 4°C, and resuspended in pre-chilled PBS. Following centrifugation, the supernatant was removed, and the cell pellet was resuspended in 1X binding buffer to achieve a density of 1 × 10^∧^6 cells/mL. The cells were then stained with 5 μL of Annexin V-FITC in the dark for a duration of 5 min at room temperature, subsequently followed by a 15-min incubation with 10 μL of PI. Apoptotic cells were assessed using a flow cytometer (FACSCanto, BD Biosciences), and the apoptosis rate (sum of upper right and lower right quadrants) was calculated utilizing FlowJo V10 software.

### 2.14 Western blotting

Cells (2 × 10^∧^6) or tissues (20 mg) were subjected to two washes with precooled PBS and lysed with 100 μL of RIPA lysis buffer. The resulting protein lysates were mixed with 5 × loading buffer and denatured by heating at 95°C for 15 min. Following this, proteins were separated using SDS/PAGE, transferred onto a PVDF membrane, and incubated overnight at 4°C with an anti-rabbit ASF1B antibody (1:1000, Proteintech). The membranes were then treated with secondary antibodies and visualized using an enhanced chemiluminescence kit.

### 2.15 Quantitative real-time PCR

Total RNA was extracted from harvested cells using TRIzol reagent (Invitrogen, CA, United States) and reverse transcribed into cDNA using a Takara kit (Shiga, Japan, #RR037A). Quantitative real-time PCR was performed using SYBR Green dye (Takara, Shiga, Japan, #RR420A) and 10 μM forward and reverse primers on a LightCycler instrument (Roche, Indianapolis, IN, United States). Primer sequences were listed.

**Table:** 

FW-ASF1B	TCC​GGT​TCG​AGA​TCA​GCT​TC
RV-ASF1B	GTC​GGC​CTG​AAA​GAC​AAA​CA
FW-GAPDH	GCA​CCG​TCA​AGG​CTG​AGA​AC
RV-GAPDH	TGG​TGA​AGA​CGC​CAG​TGG​A

### 2.16 Xenograft tumor in NSG mice

Female NSG mice, weighing 18–21 g and aged 5–6 weeks, were obtained from Shanghai Model Organism Center Co., Ltd. (NM-NSG-001). These mice were randomly assigned into two groups: CTRL and ASF1B^−/−^, with 5 mice per group. MKN-45 cells and ASF1B knockout MKN-45 cells were suspended in a mixture of phosphate-buffered saline and Matrigel matrix to prepare the cell suspensions. The cell suspension was injected subcutaneously into the NSG mice, with each mouse receiving a total of 4 × 10^∧^6 cells. Tumor growth was monitored by measuring tumor size and mouse body weight. Tumor volume (mm^∧^3) was calculated using the formula: (width^∧^2 × length)/2.

### 2.17 Human immune reconstitution

Peripheral blood mononuclear cells (PBMCs) from donors were isolated and injected into mice via the tail vein at a dose of 5 × 10^∧^6 cells. After 7 days, blood samples were collected, and red blood cells were lysed. Flow cytometry was used to evaluate the effect of human immune reconstitution.

### 2.18 Organoid culture

Fresh tumor tissues from patients were minced and washed in a medium containing 2 mM glutamine, 10% FBS, and DMEM/F12. The tissues were digested with 0.1 mg/mL collagenase IV (Roche) for 30 min at 37°C. Digestion was stopped using wash medium, and the digested tissues were filtered through a 100-μm cell strainer. The cell clusters were centrifuged, resuspended in 50% Matrigel/organoid medium, and plated. The mixture was incubated at 37°C with 5% CO_2_ for 20 min to solidify. After solidification, 0.5 mL of organoid culture medium was added, with medium changes every 3–4 days.

### 2.19 Comprehensive IP analysis for ASF1B-interacting proteins

Cells were lysed in a buffer containing Tris-HCl, NaCl, EDTA, TritonX-100, and protease inhibitors. After centrifugation at 13,000 rpm at 4°C, 50 μL of the supernatant was taken as whole cell lysate (WCL). The remaining lysate was divided and incubated with gel beads, ASF1B antibody, or control IgG at 4°C for 2 h. Beads were washed with cold lysis buffer, and proteins were eluted with elution buffer (0.1 M glycine, pH 3.5). To the protein solution, neutralization buffer (0.5 M Tris-HCl pH 7.4, 1.5 M NaCl) was added. Samples were analyzed by Western blot, with the rest frozen at −80°C. Protein spectrum analysis was performed by Novogene (Zhejiang, China).

### 2.20 Tandem mass tagging proteomics analysis

TMT quantitative proteomics was performed on ASF1B-overexpressing (OE) and control cells by Novogene (Zhejiang, China).

### 2.21 Co-immunoprecipitation (CO-IP)

HEK-293T cells infected with Flag-H2AC20 lentivirus were collected after 48 h. Cells were lysed, and supernatants collected. A 50 μL aliquot was taken as WCL, and the rest incubated with Flag beads (Sigma-Aldrich) or ASF1B antibody-conjugated gel beads at 4°C for 2 h. Beads were washed with protein lysis buffer, and samples were analyzed by Western blot to detect protein interactions.

### 2.22 Statistical analysis

Statistical analyses were performed utilizing SPSS version 24.0 (IBM) and GraphPad Prism version 8.0 (San Diego, CA, United States). Two-tailed Student’s t-tests were employed to compare two groups, while one-way ANOVA with Tamhane’s T2 or LSD *post hoc* tests was utilized for multiple group comparisons. Bioinformatics data processing was conducted using R (version 3.6.3). The data are presented as means ± standard deviation. Statistical significance is denoted as *p < 0.05, **p < 0.01, ***p < 0.001.

## 3 Results

### 3.1 ASF1B is highly expressed in gastric cancer

**FIGURE 1:**
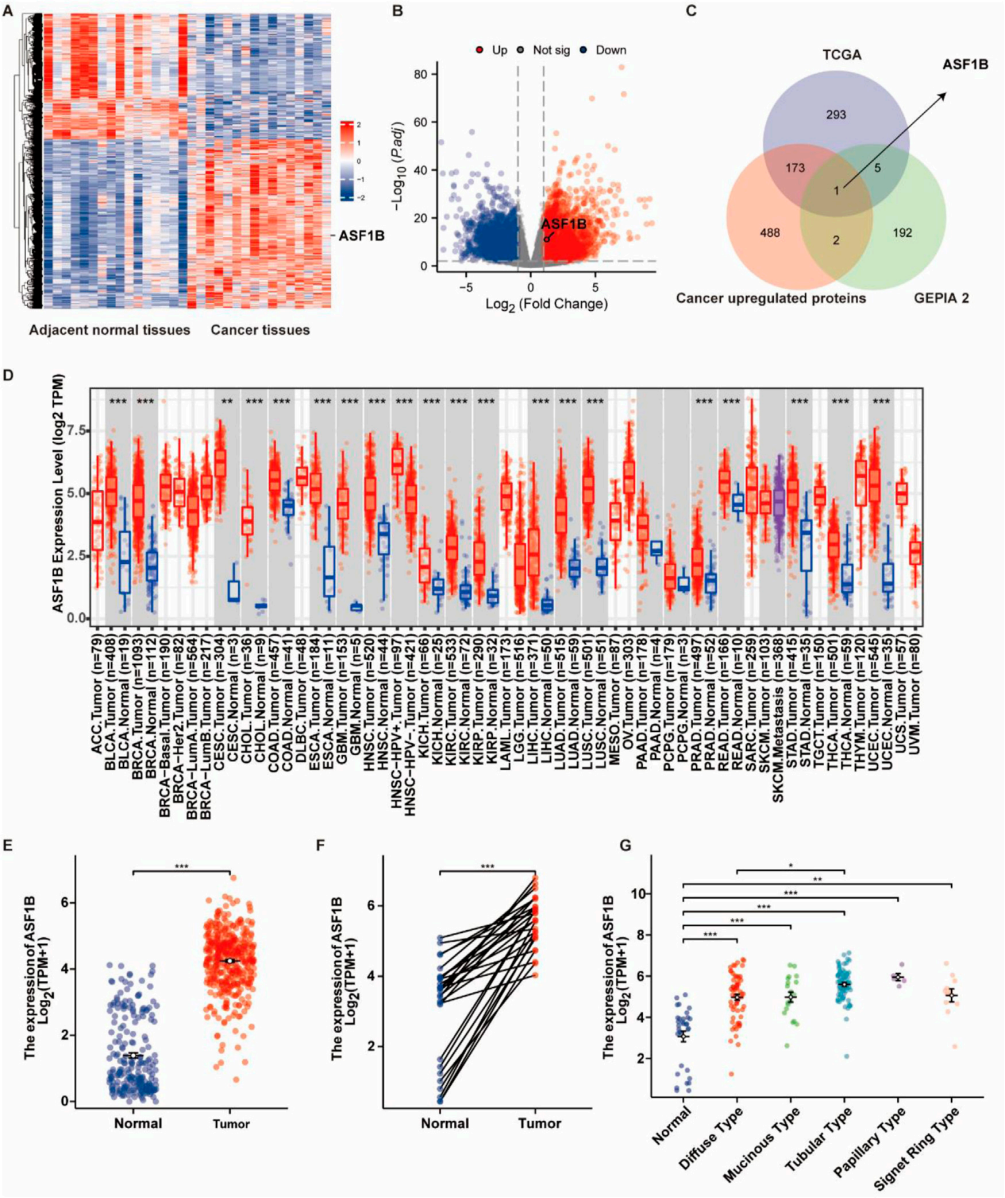
Identification of ASF1B as a highly expressed gene in gastric cancer. Microarray analysis revealed higher ASF1B expression in gastric cancer tissues compared to adjacent normal tissues **(A)**. The volcano plot highlighted differentially expressed genes **(B)**. The Venn diagram indicated overlap among genes from microarray, TCGA, and GEPIA2 databases **(C)**. TIMER2 data showed ASF1B upregulation in multiple cancers **(D)**. ASF1B was overexpressed in gastric cancer tissues according to TCGA data **(E, F)**, and differentially expressed across various histological types **(G)**.

Microarray analysis of tissues from 16 GC patients identified differentially expressed genes between tumor and adjacent normal tissues (Figure 1A). Using the DESeq2 package in R, differential gene expression in GC and normal tissues from the TCGA database was assessed. Genes with a log2 (fold change) > 1 and p < 0.05 were deemed significantly upregulated compared to normal tissues, and these were visualized in a volcano plot (Figure 1B). Survival-associated genes were obtained from the GEPIA2 database, and by comparing these with the upregulated genes in the microarray and TCGA datasets, ASF1B was identified as an overlapping gene (Figure 1C). Further analysis using TCGA and GTEx data showed that ASF1B is highly expressed in various tumors (Figure 1D). We queried the TISCH2 database for the expression levels of ASF1B in different cell types of primary human gastric cancer tumors and presented the results (Supplementary Figures S1A, B). Specifically in GC, ASF1B showed elevated expression levels in both unpaired and paired tissues (Figures 1E, F), as well as in different histological types of GC (Figure 1G). These results suggest that ASF1B is significantly upregulated in GC, indicating a potential role in tumorigenesis and progression.

### 3.2 ASF1B expression and immune cell infiltration

**FIGURE 2:**
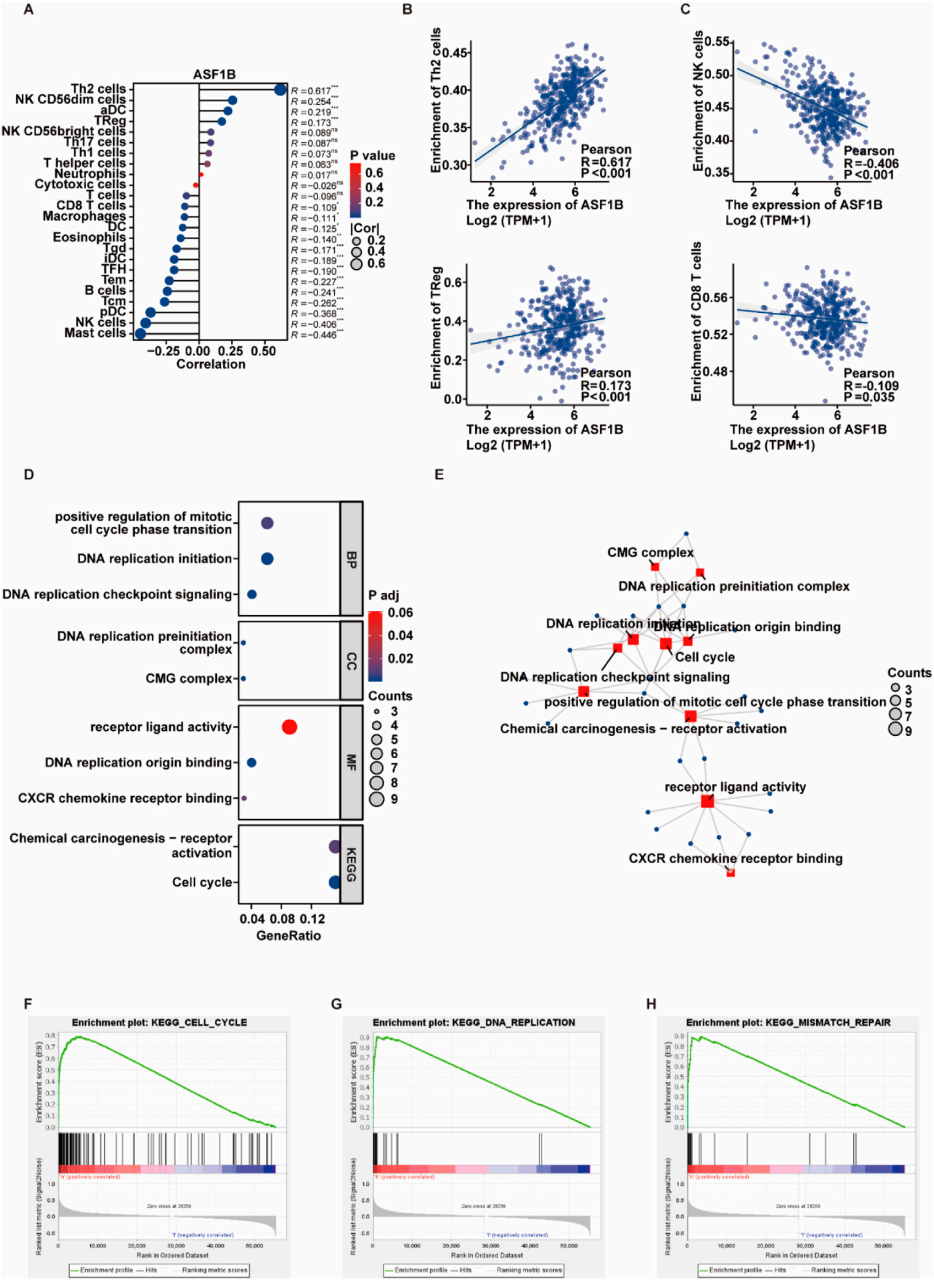
Exploring ASF1B’s relationship with immune cell infiltration and the cell cycle. A bubble plot illustrated the correlation between ASF1B expression and immune cell infiltration **(A)**. Scatter plots showed linear correlations between ASF1B expression and specific immune cells **(B, C)**. GO and KEGG pathway analyses of ASF1B-related genes **(D, E)**. GSEA showed enrichment of ASF1B mRNA expression in cell cycle and DNA replication pathways **(F, G, H)**.

ASF1B plays a significant role in the tumor microenvironment. Analysis of immune cell infiltration data sourced from the TCGA database revealed a positive correlation between ASF1B expression and the abundance of suppressive immune cells, including Th2 and Treg cells, while demonstrating a negative correlation with facilitative immune cells such as NK and CD8 T cells (Figures 2A–C).

### 3.3 ASF1B and the cell cycle

To understand ASF1B’s role in GC progression, expression profiles from the TCGA database were analyzed. GO/KEGG pathway analyses indicated that proteins upregulated in the high ASF1B expression group were predominantly associated with cell cycle and DNA replication pathways (Figures 2D, E). GSEA of mRNA expression data from the TCGA database revealed that differentially expressed genes in the high ASF1B group were enriched in cell cycle, mismatch repair, and DNA replication pathways, suggesting that ASF1B may promote cell cycle progression and DNA replication (Figures 2F–H).

### 3.4 ASF1B is highly expressed in gastric cancer tissues and correlates with poor prognosis

**FIGURE 3:**
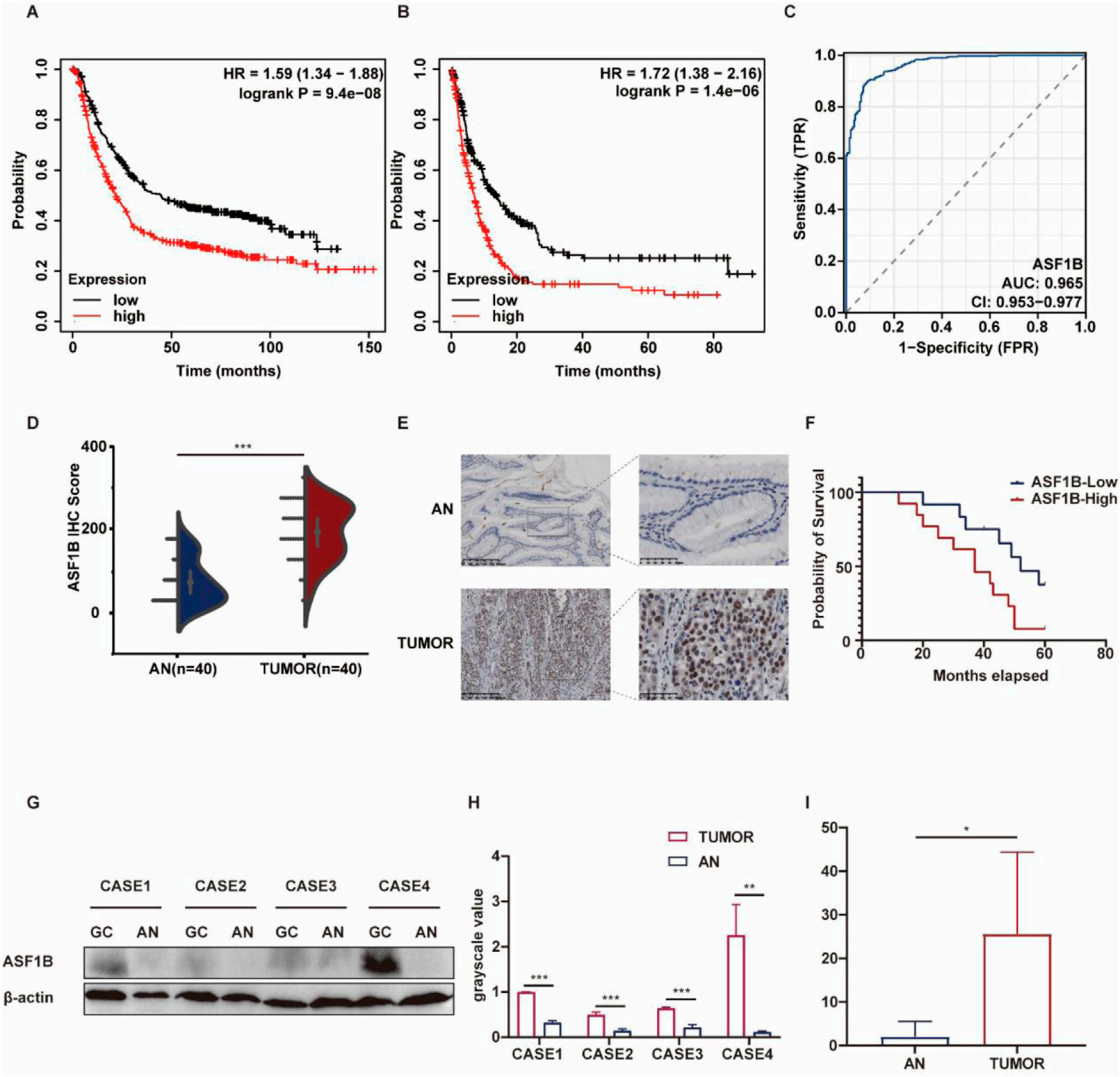
Kaplan-Meier survival curves for gastric cancer patients from the KM-plotter database **(A, B)**. ROC curve analysis for ASF1B **(C)**. Immunohistochemical results showing ASF1B expression **(D, E)**. Kaplan-Meier survival curves **(F)**. ASF1B expression detected by Western blot and qRT-PCR **(G, H, I)**.

Analysis of the KM-plotter database revealed that high ASF1B expression in patients is associated with poorer overall survival and disease-free survival (Figures 3A, B). ASF1B was also assessed as a diagnostic marker for GC, with a ROC curve AUC of 0.965 (Figure 3C). To validate ASF1B protein expression in clinical samples, immunohistochemical analysis was conducted on 40 pairs of GC and adjacent normal tissues, showing significant upregulation of ASF1B in tumor samples. This was further corroborated by Western blot analysis of tissues from 4 GC patients. A violin plot of ASF1B immunohistochemical scores from these 40 patients confirmed high expression of ASF1B in GC tissues (Figures 3D, E). Survival data from 25 GC patients, followed for over 5 years, were statistically analyzed and visualized using Kaplan-Meier survival curves (Figure 3F, *p* = 0.028). Moreover, ASF1B expression in tumor and adjacent tissues from 4 patients was confirmed to be upregulated at both protein and mRNA levels by Western blot and RT-qPCR (Figures 3G–I).

### 3.5 Clinical validation of ASF1B overexpression and construction of ASF1B-Knockout gastric cancer cell lines

**FIGURE 4:**
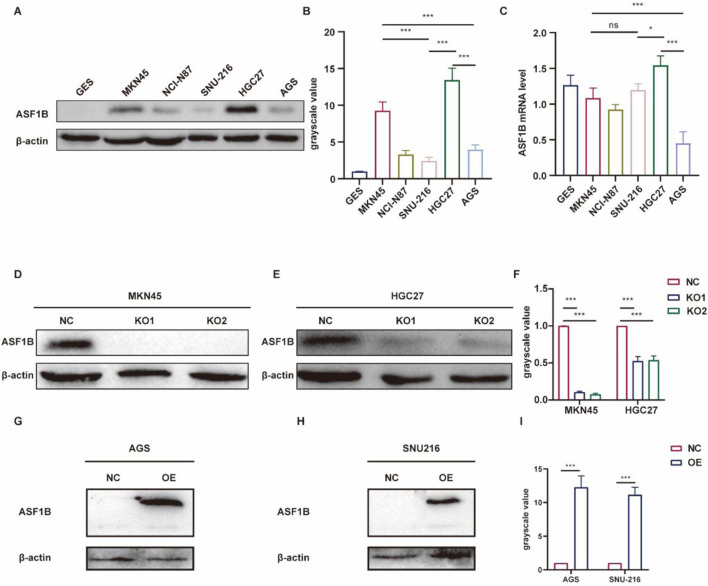
ASF1B expression in MKN45 and HGC27 cell lines was confirmed among six gastric cancer cell lines via WB **(A)**. WB results were quantified and represented in histograms **(B)**. qRT-PCR results for the 6 cell lines **(C)**. Knockout efficiency of two lentiviruses with different guide RNAs in MKN45 cells was assessed by WB **(D)**, and similarly in HGC27 cells **(E)**. WB results were quantified and shown in histograms **(F)**. ASF1B overexpression efficiency was determined by WB **(G, H)**, with quantification shown in histograms **(I)**.

To explore ASF1B’s role in GC, we first identified MKN45 and HGC27 cell lines as ASF1B-overexpressing through Western blot (WB) and qRT-PCR analysis (Figures 4A–C). Subsequently, ASF1B-knockout lines, MKN45^−/−^ and AGS^−/−^, were created (Figures 4D–F). Additionally, ASF1B knock-in cell lines were developed in AGS and SNU216 cells, which initially had low ASF1B expression (Figures 4G–I). The efficiency of these modifications was verified by WB, and the modified cell lines were utilized in subsequent experiments.

### 3.6 Biological function of ASF1B in gastric cancer

**FIGURE 5:**
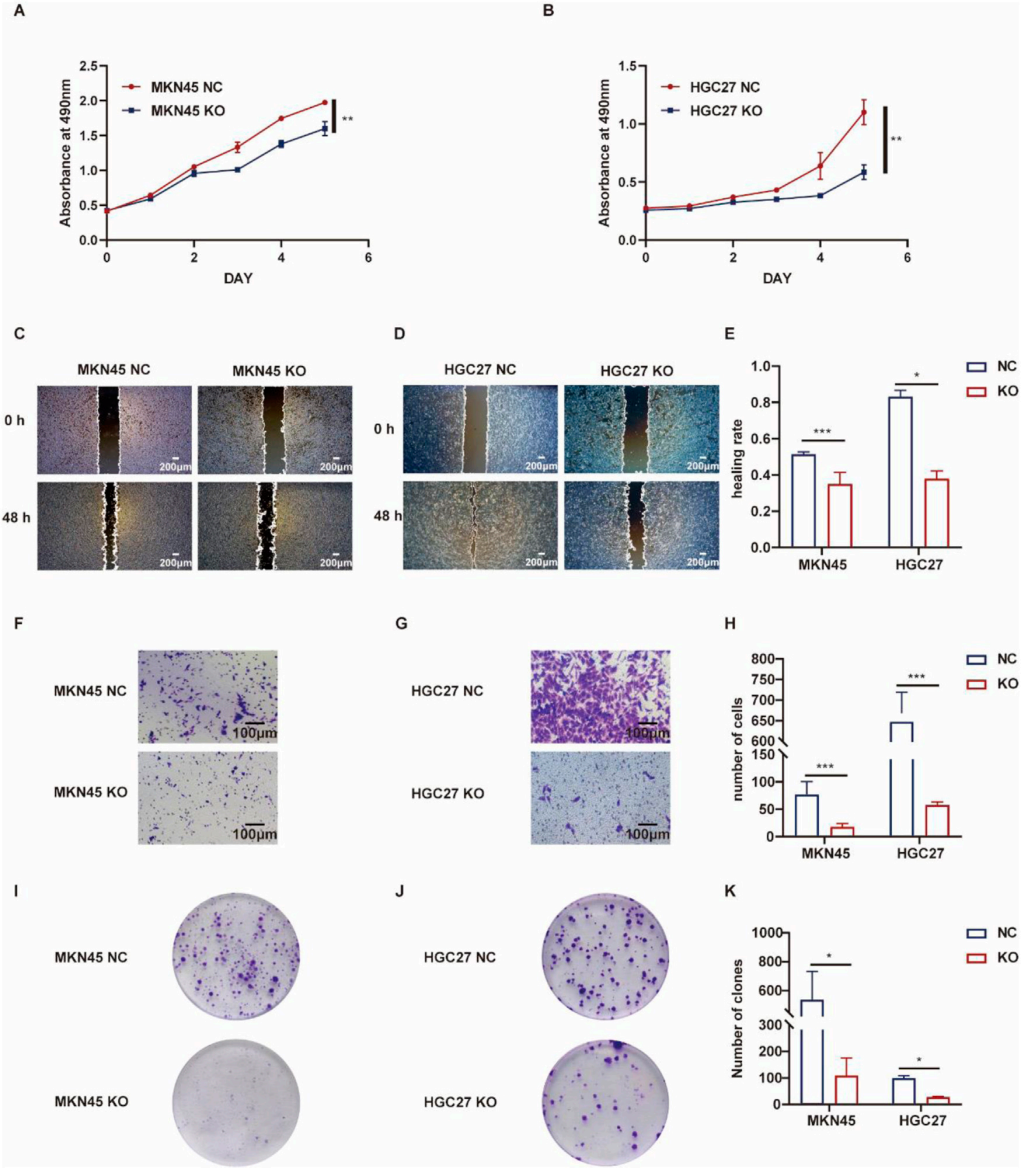
Proliferation of MKN45 cells and ASF1B-knockout cells was monitored over 5 days, with the resulting growth curves presented **(A, B)** (n = 3). Scratch assays and statistical analysis for AGS and ASF1B-knockout cells **(C, D)** (n = 3). Transwell invasion assays and analyses for MKN45 and ASF1B-knockout cells **(F, G)** (n = 3). Colony formation and statistical analysis for AGS and ASF1B-knockout cells after 3 days of culture **(I, J)** (n = 3). Statistical results are shown in histograms **(E, H, K)** (n = 3).

The role of ASF1B in gastric cancer was explored using both knockout and overexpression cell lines. Compared to ASF1B-knockout cells, control cells exhibited significantly faster proliferation (Figures 5A, B). In scratch assays, ASF1B-knockout cells showed markedly reduced migratory abilities compared to controls (Figures 5C, D). Transwell invasion assays revealed that ASF1B knockout significantly lowered the number of MKN45 and HGC27 cells invading the lower chamber (Figures 5F, G). Furthermore, colony formation assays demonstrated that ASF1B knockout notably diminished clonogenic potential in these cell lines (Figures 5I, J). Statistical analysis results are depicted in histograms (Figures 5E, H, K). These findings consistently indicate that ASF1B knockout leads to significant inhibition of gastric cancer cell proliferation.

**FIGURE 6:**
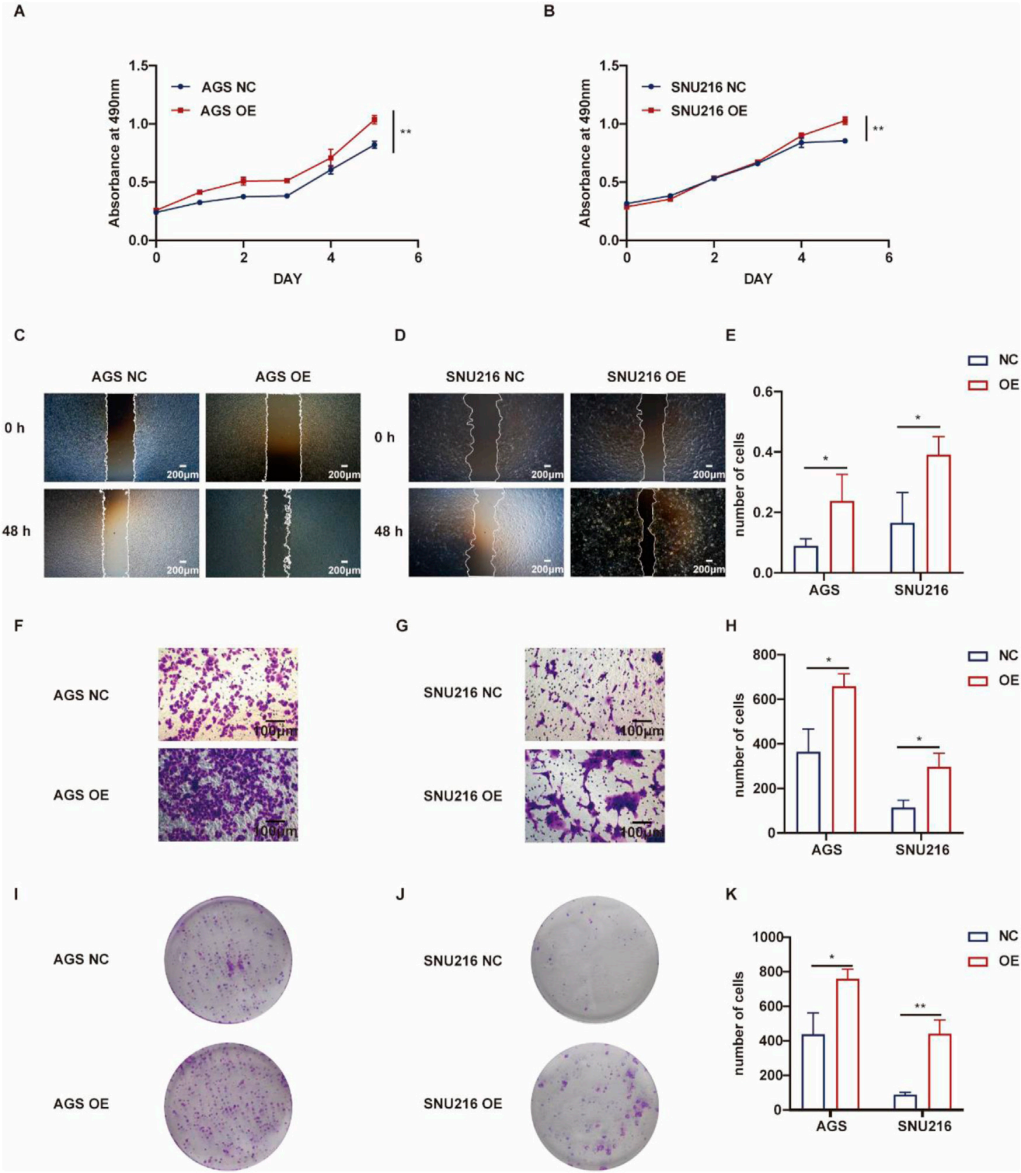
Proliferation of AGS cells and ASF1B-overexpressing cells was tracked for 5 days, with growth curves displayed **(A, B)** (n = 3). Scratch assays and statistical analyses for AGS and ASF1B-overexpressing cells **(C, D)** (n = 3). Transwell invasion assays and analyses for AGS and ASF1B-overexpressing cells **(F, G)** (n = 3). Colony formation and statistical results for AGS and ASF1B-overexpressing cells after 14 days of culture **(I, J)** (n = 3). Statistical analysis is presented in histograms **(E, H, K)** (n = 3).

Conversely, gastric cancer cells with ASF1B overexpression demonstrated enhanced proliferation compared to controls (Figures 6A, B). Scratch assays indicated that ASF1B-overexpressing cells had a significantly faster healing rate (Figures 6C–E). In transwell invasion assays, ASF1B overexpression substantially increased the invasiveness of AGS and SNU216 cells (Figures 6F, G). Similarly, ASF1B overexpression significantly boosted colony formation and clonogenic ability in these cells (Figures 6I, J). Statistical analysis results are depicted in histograms (Figures 6E, H, K). Overall, these experiments confirm that ASF1B overexpression significantly promotes gastric cancer cell proliferation.

### 3.7 Effects of ASF1B on cell cycle and apoptosis

**FIGURE 7:**
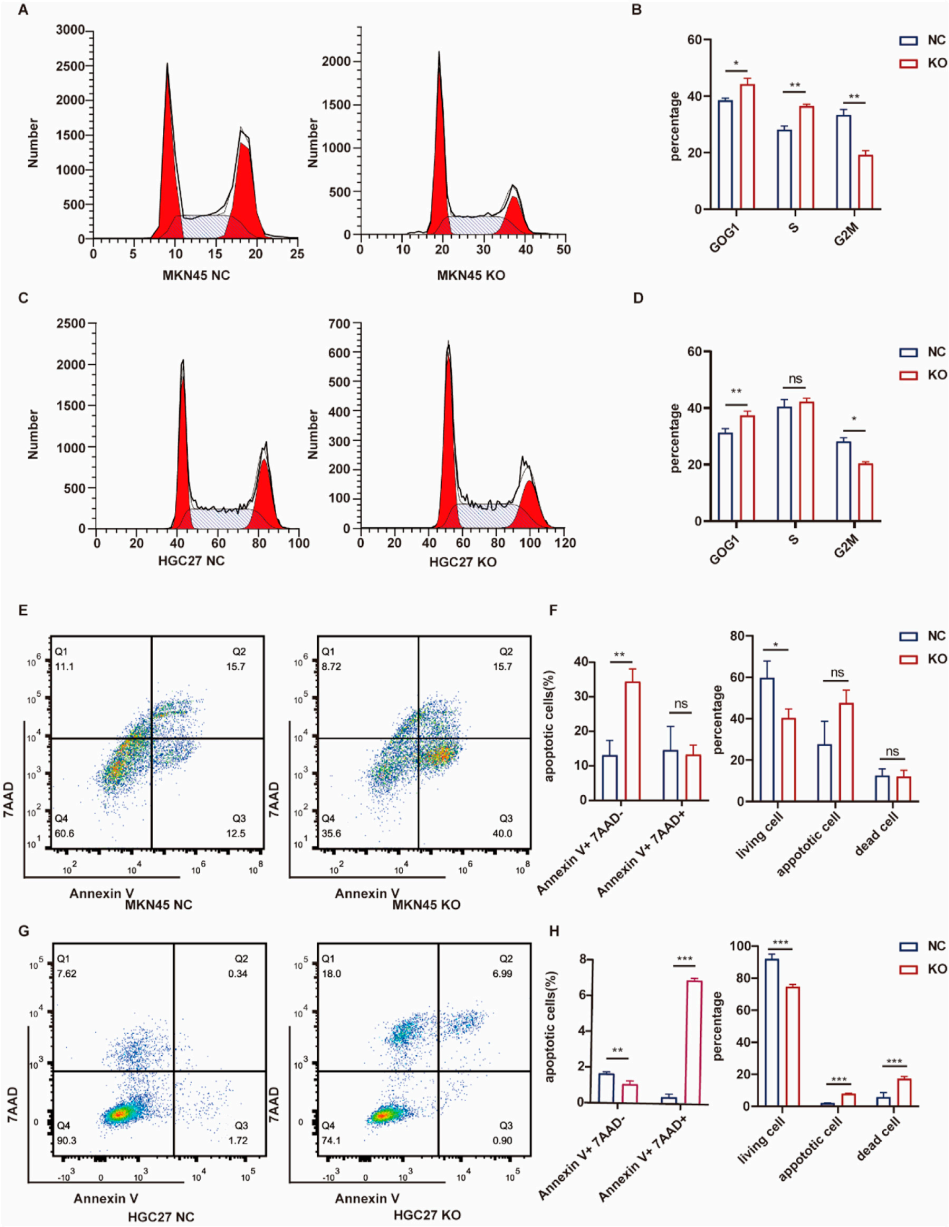
Flow cytometry analysis of the cell cycle and statistical results for MKN45 and ASF1B-knockout cells **(A, B)** (n = 3). Cell cycle analysis and statistics for HGC27 and ASF1B-knockout cells **(C, D)** (n = 3). Apoptosis analysis and statistical results for MKN45 and ASF1B-knockout cells **(E, F)** (n = 3). Apoptosis analysis and results for HGC27 and ASF1B-knockout cells **(G, H)** (n = 3).

ASF1B’s involvement in tumor cyclin regulation was investigated. Flow cytometry analysis revealed that ASF1B knockout resulted in a higher proportion of cells being arrested in the G0/G1 phase, supporting the GSEA findings (Figures 7A, C). ASF1B appears to facilitate the transition from the G0/G1 phase to the G2/M phase through interactions with cyclins. Additionally, flow cytometry indicated that ASF1B knockout promotes apoptosis in gastric cancer cells (Figures 7E, G). Statistical analysis results are presented in histograms (Figures 7B, D, F, H).

**FIGURE 8:**
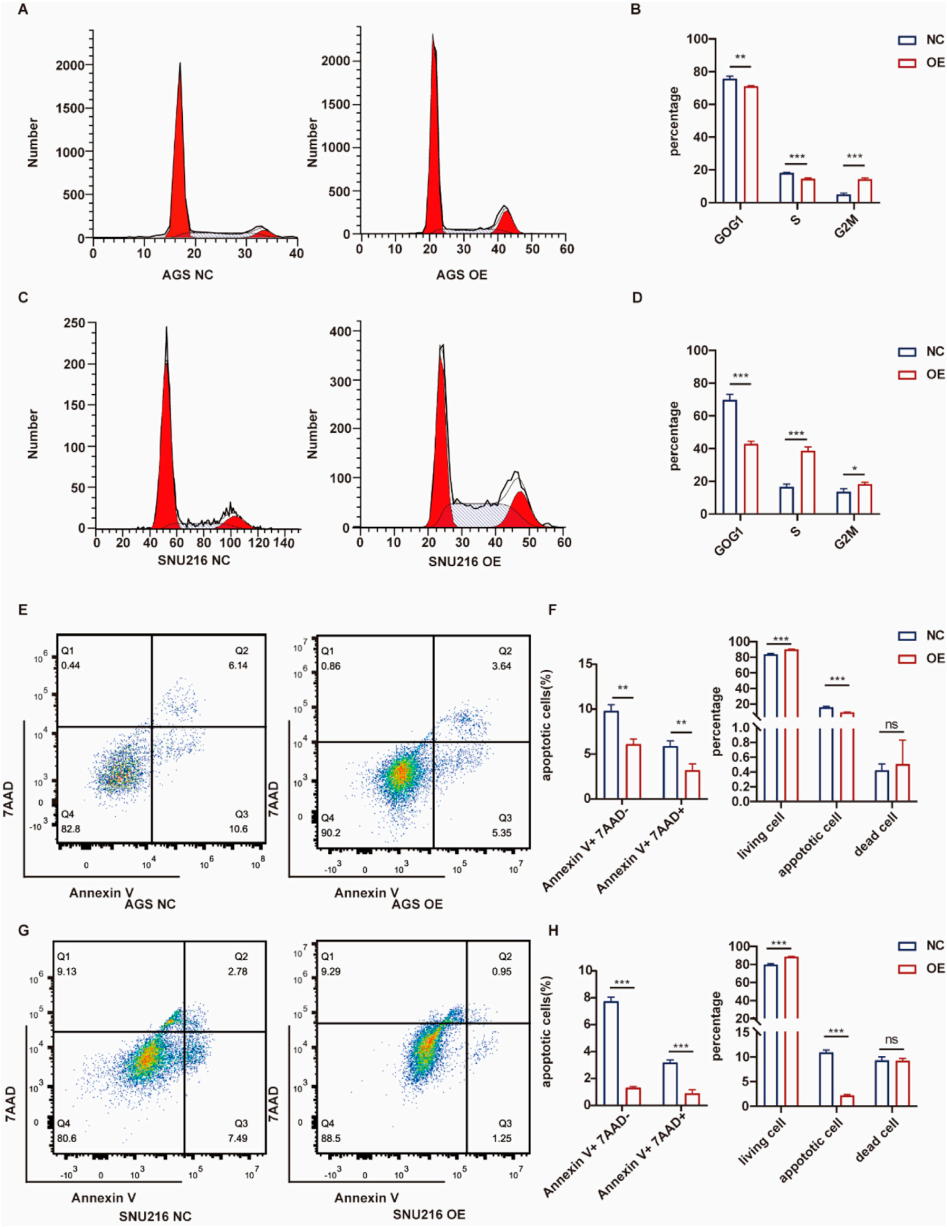
Flow cytometry analysis and statistical results for AGS and ASF1B-overexpressing cells **(A, B)** (n = 3), and for SNU216 and ASF1B-overexpressing cells **(C, D)** (n = 3). Apoptosis analysis and statistical results for AGS **(E, F)** and SNU216 **(G, H)** cells with ASF1B overexpression (n = 3).

Flow cytometry was employed to analyze the distribution of cells across different cell cycle phases. Overexpression of ASF1B in gastric cancer cells resulted in a reduced proportion of cells arrested in the G0/G1 phase, consistent with GSEA findings (Figures 8A, C). ASF1B facilitates the cell cycle transition from G0/G1 to G2/M by interacting with cyclins. Additionally, flow cytometry revealed that ASF1B overexpression inhibited apoptosis (Figures 8E, G). The statistical analysis results are displayed in histograms (n = 3) (Figures 8B, D, F, H).

### 3.8 ASF1B knockout inhibits subcutaneous tumor growth and enhances CD8^+^ T cell infiltration in NSG mice

**FIGURE 9:**
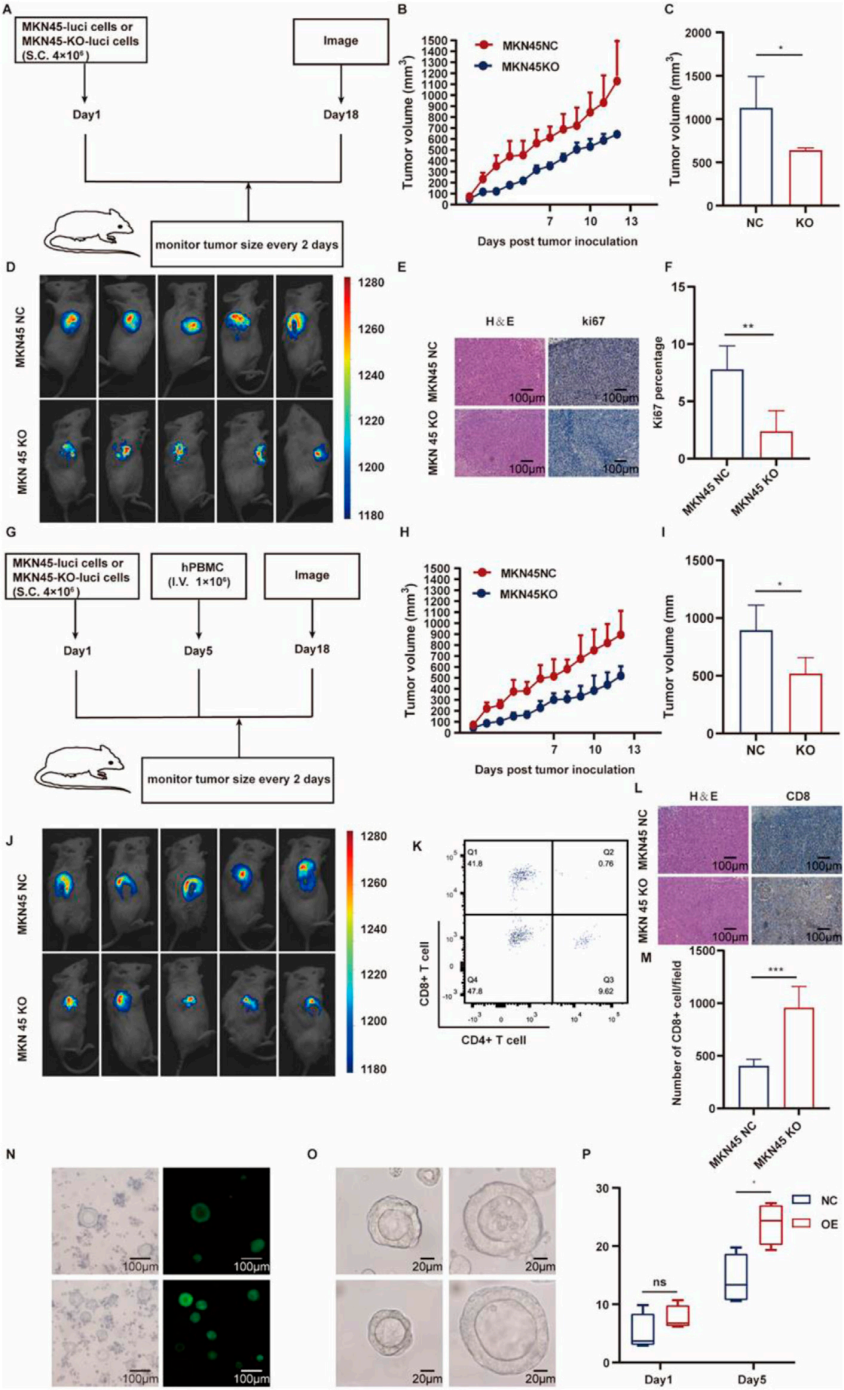
Schematic representation of the animal experiment procedure **(A)** (n = 3). Tumor growth curves for NSG mice in the two groups **(B, C)** (n = 3). Day 13 images of subcutaneous tumors in NSG mice from both groups **(D)** (n = 3). IHC analysis for KI67 expression in tumors **(E, F)** (n = 3). Diagram illustrating the procedure for human immune reconstitution **(G)** (n = 3). Tumor growth curves following immune reconstitution **(H, I)** (n = 3). Day 13 images of subcutaneous tumors post-reconstitution **(J)** (n = 3). Flow cytometry results for human immune reconstitution effects in mouse blood **(K)**. IHC analysis for CD8 expression in tumors **(L, M)** (n = 3). Construction of ASF1B-overexpressing patient-derived organoids **(N)**. Organoid volumes after 5 days in different groups **(O, P)** (n = 4).

To explore the *in vivo* impact of ASF1B on tumor growth, a subcutaneous transplantation model was created by injecting ASF1B-knockout or control MKN45 cells into NSG mice (Figure 9A). Tumors were visualized via *in vivo* imaging on day 13 post-injection (Figure 9D), and subcutaneous tumor volume was tracked and plotted (Figures 9B, C). ASF1B knockdown reduced the expression of the proliferation marker Ki67 in gastric cancer tissues (Figures 9E, F).

Human immune reconstitution was performed in NSG mice, and the experiments were repeated (Figure 9G). Tumor volume changes were monitored, with imaging conducted (Figures 9G–I). Flow cytometry was used to assess the effects of human immune reconstitution on mouse blood (Figure 9K). ASF1B knockdown led to increased CD8^+^ T cell infiltration (Figures 9L, M). These *in vivo* results suggest that ASF1B knockout inhibits subcutaneous tumor formation by MKN45 cells in NSG mice.

### 3.9 Organoids with ASF1B overexpression show accelerated proliferation

Patient-derived gastric cancer organoids were generated, with ASF1B overexpressed. Overexpression efficiency was confirmed via fluorescence microscopy (Figure 9N). Organoid volume changes were recorded and depicted in a line graph (Figures 9O, P). After 5 days, ASF1B-overexpressing organoids demonstrated significantly larger volumes.

### 3.10 ASF1B interacts with H2AC20 in gastric cancer

**FIGURE 10:**
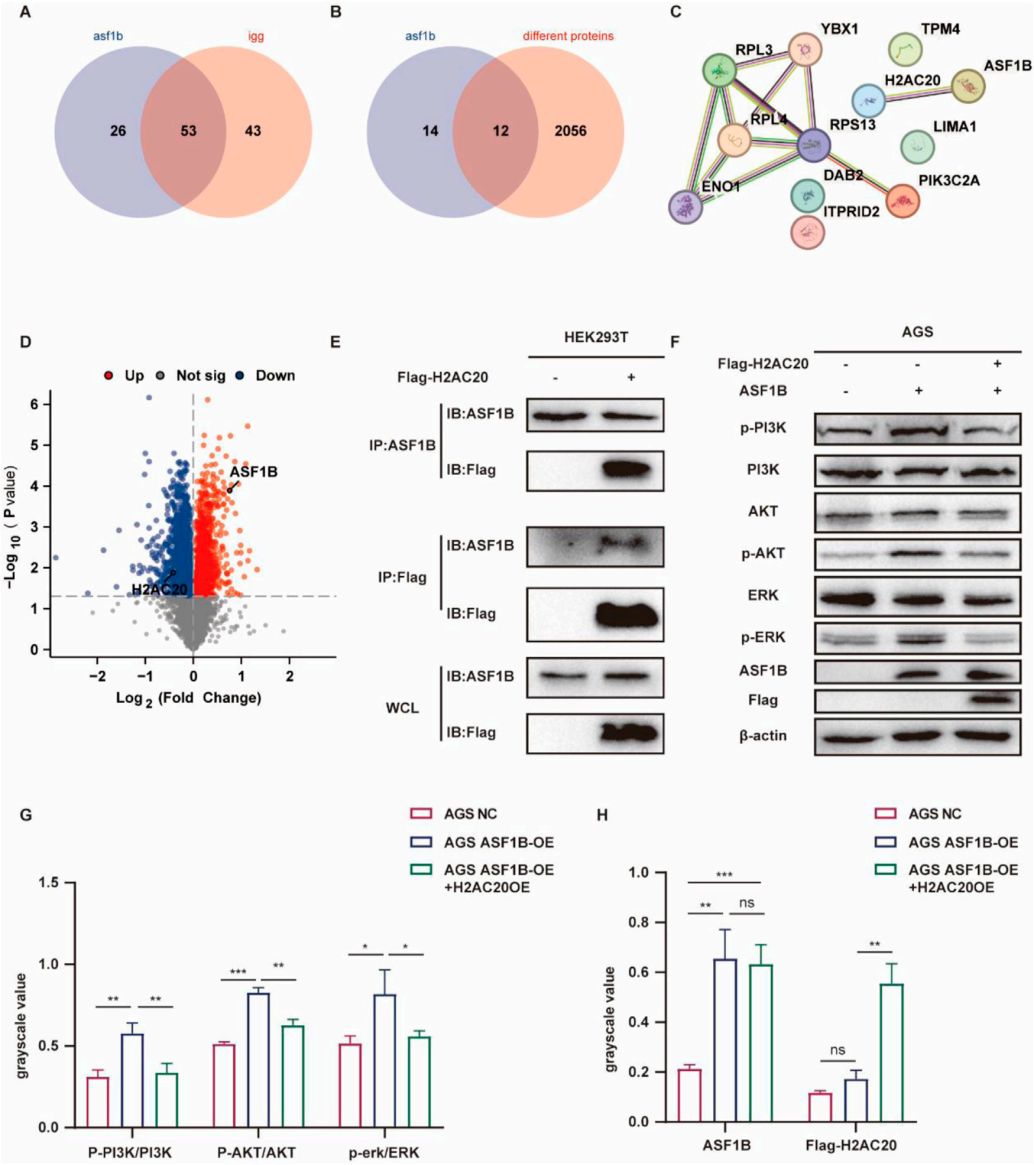
Protein spectrum analysis of ASF1B-captured proteins **(A)**. Comparison of TMT-identified proteins with protein spectrum data **(B)**. String database analysis of potential ASF1B interactors **(C)**. Volcano plot showing H2AC20 downregulation in ASF1B-overexpressing cells **(D)**. CO-IP detection of ASF1B and H2AC20 in HEK293T cells **(E)**. Overexpression of H2AC20 in ASF1B-overexpressing gastric cancer cells with subsequent WB detection of PI3K/AKT and ERK1/2 pathway proteins **(F, G, H)**.

In ASF1B-overexpressing AGS cells, proteins bound by the ASF1B antibody were analyzed via mass spectrometry, identifying 26 proteins that specifically interact with ASF1B (Figure 10A). The TMT experiment revealed 2068 differentially expressed proteins, with 12 proteins identified as potential ASF1B binders displaying altered expression levels (Figure 10B). String database analysis indicated that H2AC20 is a likely binding partner of ASF1B (Figure 10C). The expression of H2AC20 was significantly decreased in ASF1B overexpressed cell lines (Figure 10D). And the expression of H2AC20 was significantly increased in ASF1B knockout cell lines (Supplementary Figure S1D). This interaction was confirmed through co-immunoprecipitation (Figure 10E; Supplementary Figure S1C).

### 3.11 H2AC20 inhibits ASF1B’s biological function

**FIGURE 11:**
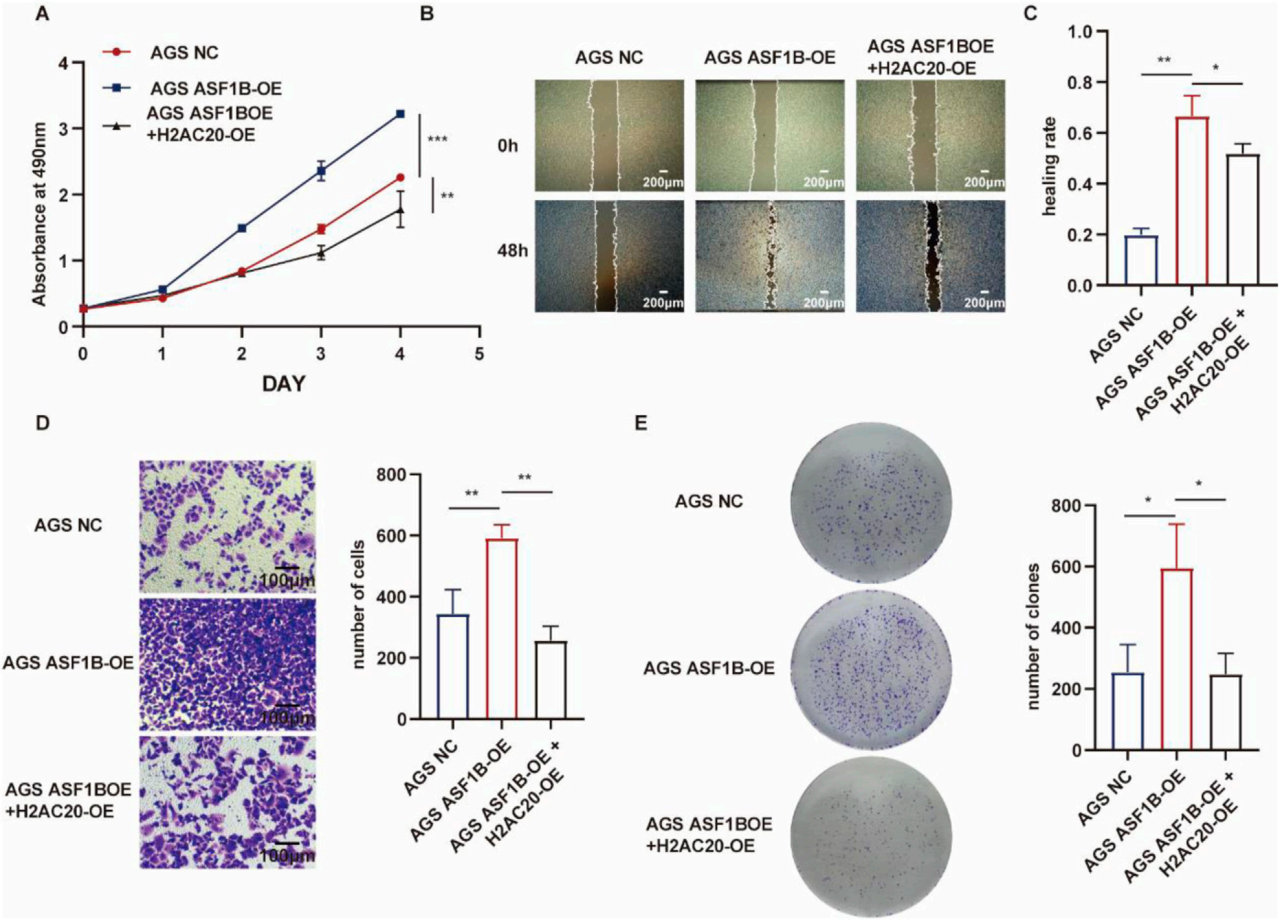
MTT assay monitoring proliferation of AGS cells, ASF1B-overexpressing cells, and H2AC20-overexpressing ASF1B cells over 5 days **(A)** (n = 3). Scratch assay and statistical analysis of AGS cells, ASF1B-overexpressing cells, and H2AC20-overexpressing ASF1B cells **(B, C)** (n = 3). Transwell invasion assay and statistical analysis of AGS cells, ASF1B-overexpressing cells, and H2AC20-overexpressing ASF1B cells **(D)** (n = 3). Colony formation and statistical analysis for AGS cells, ASF1B-overexpressing cells, and H2AC20-overexpressing ASF1B cells after 14 days of culture **(E)** (n = 3).

H2AC20 was overexpressed in ASF1B-overexpressing gastric cancer cells. In our previous study, overexpression of ASF1B in AGS cells promoted the expression of phosphorylated AKT, phosphorylated PI3K, and phosphorylated ERK (Supplementary Figure S1E), and H2AC20 could reverse this process (Figures 10F–H). ASF1B overexpression accelerated wound healing (Figure 11A) and increased AGS cell invasion in Transwell assays (Figure 11D). ASF1B also enhanced colony formation in AGS cells (Figure 11E). WB analysis showed that ASF1B activated the PI3K/AKT and ERK1/2 pathways, effects that were reversed by H2AC20 overexpression (Figures 11A–E). These results consistently demonstrate that H2AC20 can counteract ASF1B-induced proliferation and invasion in gastric cancer cells. Statistical analysis results are shown in the histograms below.

### 3.12 H2AC20 reverses ASF1B-induced cell cycle progression and apoptosis inhibition

**FIGURE 12:**
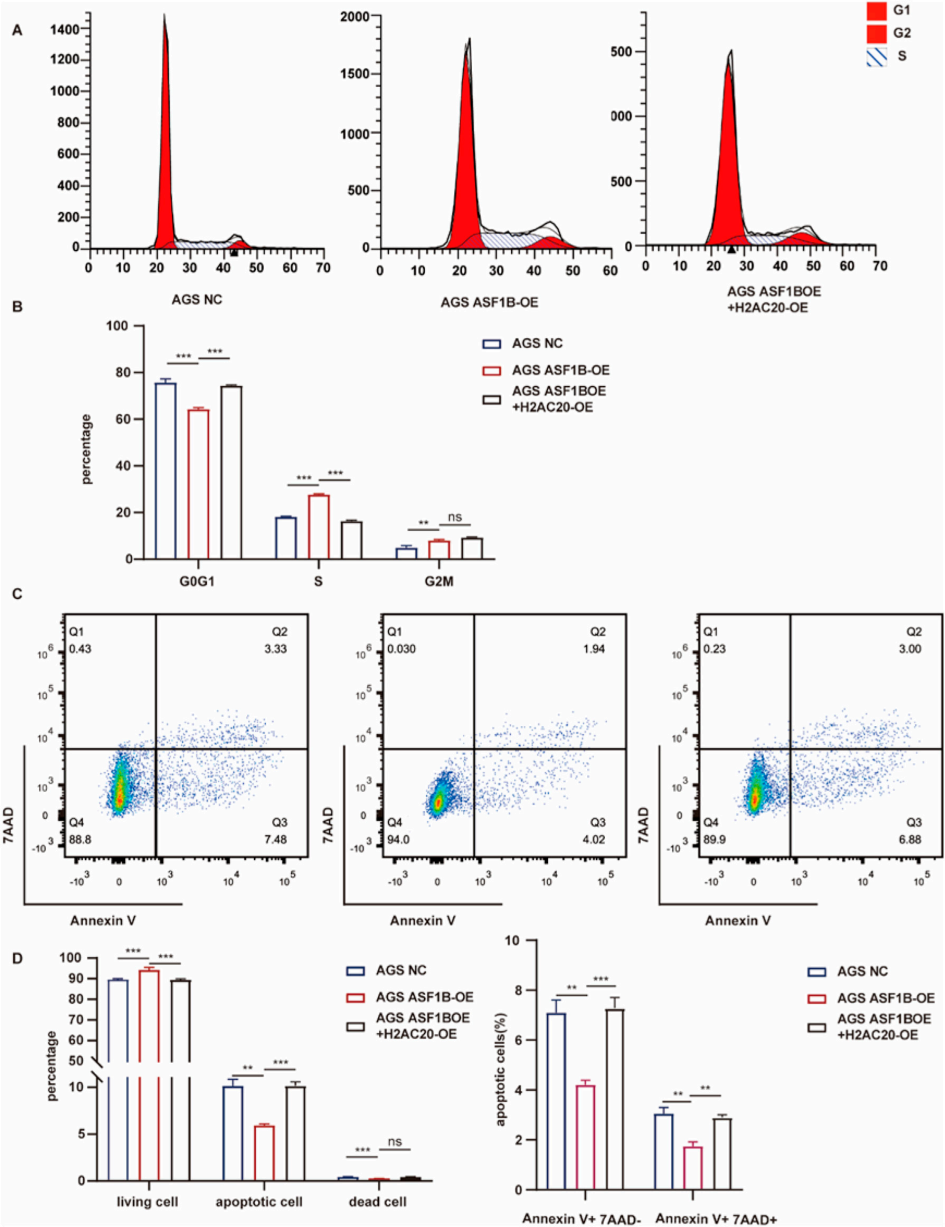
Flow cytometry analysis and statistical results for AGS cells, ASF1B-overexpressing cells, and H2AC20-overexpressing ASF1B cells **(A, B)** (n = 3). Flow cytometry and statistical analysis of apoptosis for the same cell lines **(C, D)** (n = 3).

ASF1B overexpression in gastric cancer cells promotes cell cycle progression and inhibits apoptosis. Flow cytometry analysis showed that H2AC20 overexpression reversed the effect of ASF1B on the cell cycle (Figure 12A). Similarly, H2AC20 overexpression counteracted ASF1B’s inhibition of apoptosis (Figure 12C). Statistical analysis results are presented in histograms (Figures 12B, D).

## 4 Discussion

Gastric cancer poses a significant threat to public health, ranking among the most prevalent and lethal malignancies worldwide (Sung et al., 2021). The management of GC presents numerous challenges due to its unfavorable prognosis and notable resistance to chemotherapy in advanced stages. However, with the ongoing development of treatment modalities, the field of GC has transitioned into an era characterized by comprehensive management driven by precision and standardization. Notably, precision medicine—particularly gene-targeted therapies—presents considerable potential. Identifying viable therapeutic targets for GC remains a critical area of investigation. By integrating analyses of tissue microarray data with the TCGA and GEPIA2 databases, we pinpointed the pivotal gene ASF1B. Our research revealed that ASF1B expression was significantly elevated in GC, showing high expression across various gastric cancer subtypes. Statistically, we conducted immunohistochemical assessments of ASF1B in 40 pairs of cancerous and adjacent normal tissues, subsequently analyzing survival outcomes for 25 GC patients followed for over 5 years. The findings indicated that ASF1B expression was markedly higher in GC tissues and correlated with poor prognostic outcomes. This implies its potential role in oncogenesis and the progression of GC. These results align with prior research findings (Zhao et al., 2024; Zhang et al., 2023). As an oncogene, ASF1B has demonstrated the capability to facilitate the growth of other solid tumors, with its expression levels closely associated with survival prognosis (Misiewicz-Krzeminska et al., 2013; Wang et al., 2020; Zhang and Liu, 2023; Corpet et al., 2011; Chen et al., 2020; Carrion et al., 2020; Zhang et al., 2024; Xu et al., 2019; Liu et al., 2020; Zhang et al., 2021).

To further clarify the effect of ASF1B on the biological characteristics of GC cells, we used CRISPR-case9 gene editing technology to construct MKN45 and HGC27 gastric cancer cell ASF1B^−/−^ knockout cell lines and constructed AGS and HGC27 GC cell ASF1B^+/+^ overexpression cell lines using ASF1B overexpression vectors. Based on these works, biological function experiments were conducted, proving that ASF1B contributes to tumor cell proliferation, clone formation, invasion, and migration *in vitro*. *In vivo*, ASF1B knockout suppressed the subcutaneous tumorigenicity of MKN-45 cells. In the constructed patient-specific cancer organoids, ASF1B-overexpressing organoids showed faster proliferation levels. We confirmed the promotional impact of ASF1B on gastric cancer growth through *in vivo*, *in vitro*, and organoid studies. Furthermore, we analyzed GC data from the TCGA database, indicating that ASF1B may act as an accelerator of the cell cycle and DNA replication. These findings were corroborated by flow cytometry analysis. ASF1B facilitates the progression of the cell cycle from the G0/G1 phase to the G2/M phase and inhibits apoptosis. These results substantiate the previously documented tumor-promoting function of ASF1B in GC (Zhao et al., 2024; Zhang et al., 2023).

Investigations have previously established an obvious relationship between ASF1B expression and immune cell infiltration. ASF1B is linked to multiple categories of immune infiltrating cells and immune signaling pathways, thereby impacting tumor progression through modifications in immune infiltration dynamics. Zhang et al. (2022a) and Zhan et al. (2021) found that the transcription levels of ASF1B exhibited a positive correlation with the extent of immune cell infiltration in hepatocellular carcinoma (HCC), including CD4^+^ T cells, dendritic cells (DCs), neutrophils, CD8^+^ T cells, and B cells. In stomach adenocarcinoma (STAD) (Im et al., 2016), Zhao et al. found a significant positive correlation between ASF1B expression and the infiltration levels of aDC cells, CD56dim cells, Th2 cells, Th17 cells, and TReg cells. Conversely, they observed a negative correlation with the infiltration levels of B cells, mast cells, NK cells, pDCs, Tcm, Tem, and TFH cells. Moreover, STAD patients exhibiting elevated ASF1B expression tend to have higher PD-1 Immunophenoscore (IPS) and could potentially derive greater benefits from immunotherapeutic interventions. However, the correlation between ASF1B expression and the abundance of immune cell infiltration was significantly different in lung adenocarcinoma (LUAD) (Song et al., 2024). Song et al. observed that in LUAD, there was an increased abundance of infiltration from 7 cell types in the low ASF1B group, which included B cells, DCs, iDCs, mast cells, neutrophils, tumor-infiltrating lymphocytes (TILs), and T helper cells. In contrast, the high ASF1B group exhibited elevated infiltration levels of CD8^+^ T cells, NK cells, and Th1 cells. The results suggest that ASF1B may serve distinct functions related to immune response within diverse cancer microenvironments, underscoring the complexity and variability of tumor ecosystems. Our research further established that ASF1B is influential in the gastric cancer tumor microenvironment through comprehensive bioinformatics analyses and *in vivo* studies. Firstly, our bioinformatics analysis revealed a positive correlation between the expression of ASF1B and the enrichment of inhibitory immune cells, such as Th2 and Treg cells, while indicating a negative correlation with the infiltration of promoting immune cells, such as NK and CD8 T cells. This is consistent with the findings of Zhao et al. (Im et al., 2016) in STAD. Then building on this foundational data, we conducted validation experiments in human-immune-reconstituted NSG mice, which confirmed that the knockdown of ASF1B enhanced CD8^+^ T cell infiltration. The current paradigm in immunotherapy focuses on revitalizing CD8^+^ T cell responses (Singh et al., 2013). Our research findings suggest that ASF1B could serve not only as a prognostic biomarker but also as a potential therapeutic target for GC. Patients with GC exhibiting high levels of ASF1B expression may benefit from immunotherapy. Notably, while our data preliminarily validates the significance of ASF1B in GC, the underlying mechanisms require further investigation.

We identified 12 proteins that potentially bind with ASF1B and alter expression levels through comprehensive IP analysis and TMT quantitative proteomics analysis. These proteins were analyzed using the String database. It was found that H2AC20 might be a protein that specifically binds to and interacts with ASF1B. The interaction between them was confirmed by endogenous immunoprecipitation. H2AC20(also named Hist2h2ac) encodes a canonical histone isoform of histone H2A, H2A type 2-C (H2A 2C). In the past, core histone isoforms were considered to be functionally identical entities, and there was a notable absence of comprehensive research on the subject. In 2013, Singh et al. (2015) discovered that replication-dependent histone isoforms can have different cellular functions and that the regulation of these isoforms may play a role in cancers. The levels of some replication-dependent H2A isoform genes may be significantly up- or downregulated in tumor tissue samples. Further research by Rajbir Singh et al. (Monteiro et al., 2017) revealed that as bladder cells become more invasive, unmodified H2A 2C and acetylated/methylated forms of H2A 1J showed decreased expression levels. Fatima Liliana Monteiro et al. (Zhang et al., 2022b) discovered that Hist2h2ac is a novel regulatory factor involved in the proliferation and epithelial-mesenchymal transition of mammary epithelial and breast cancer cells, playing an oncogenic role in breast cancer. During the differentiation process of mammary epithelial cells, the expression of Hist2h2ac is regulated at the transcriptional level by activating the MEK 1/2 or PI3-K pathways. Hist2h2ac acts downstream of EGFR, supporting a positive feedback loop. Zhang et al. (Chen et al., 2022) reported that ubiquitination of histone H2A is a marker of aging and pan-cancer prognosis. The transcription of H2A’ E3s/DUBs alter with aging, and the ubiquitination of H2A, including the H2AC20 isoform, is significantly regulated. The occurrence and progression of tumors are closely associated with the aging process. Different expression subtypes of H2A’ E3s/DUBs exhibit varying prognostic implications, DNA damage response (DDR) characteristics, and levels of cellular infiltration within the tumor microenvironment. We overexpressed H2AC20 in ASF1B-OE gastric cancer cells and discovered that H2AC20 can reverse the promoting effects of ASF1B on gastric cancer cell proliferation and invasion, as well as the influence of ASF1B on the cell cycle and apoptosis. The results suggest a potential tumor-suppressive role in the development and progression of gastric cancer. The performance of H2AC20 in gastric cancer is consistent with previous findings in bladder cells, but different from that in breast cancer. Whether this is related to the different roles of H2AC20 in different cancer backgrounds and at different stages of cancer development, existing studies have not given a clear explanation and further investigation is needed.

Finally, we assessed the expression levels of proteins associated with the PI3K/AKT and ERK1/2 signaling pathways through WB analysis. The results indicate that the overexpression of ASF1B can activate these pathways, while the activation is reversed when H2AC20 is overexpressed. Chen et al. (Mafi et al., 2021) found that knockout of ASF1b reduced the phosphorylation levels of PI3K and AKT in AGS and MGC803 cells. Our results are consistent with them. It is well known that the activation of PI3K/AKT/mTOR (PAM) signaling pathway plays a key role in cell growth, proliferation and cell repair. Recent studies have found that it also plays an important role as a key regulator in the tumor microenvironment. Sahar Mafi et al. (Lastwika et al., 2016) found that PAM inhibition in the nutrient-restricted tumor microenvironment could disrupt the balance between CD4+/CD8+ T cells, T lymphocytes/Th17 cells, and M1/M2 macrophages due to its role in regulating PAM metabolism. This may be a potential mechanism by which ASF1B regulates the tumor microenvironment of GC. Furthermore, many studies (Mittendorf et al., 2014; Song et al., 2013; Gao et al., 2018) have linked activated PAM signaling to increased PD-L1 expression. Mittendorf (Song et al., 2013) and Song et al (Gao et al., 2018). have demonstrated that PAM inhibition can reduce PD-L1 expression in PTEN-driven breast and CRC cells. Gao’s research (Guo et al., 2020) in lung cancer has revealed that inhibiting PI3K can downregulate PD-L1 expression and enhance the antiproliferative effects of IFN-γ, indicating that blocking PI3K can maximize the antitumor effects mediated by IFN-γ. The ERK signaling pathway is a crucial component of the RAF-MEK-ERK signaling cascade, widely present in human cancers. The hyperactivation of ERK leads to the sustained activation of its downstream substrates, resulting in the proliferation, differentiation, and metastasis of tumor cells (Shafei et al., 2020). The PAM pathway is often cross-regulated with the Ras/ERK pathway (Serra et al., 2011). Earlier, Serra et al. (Hu et al., 2021b) discovered that using ErbB-2 inhibitors or MEK inhibitors in conjunction with PI3K inhibitors enhances the efficacy of the treatment, leading to reduced proliferation of breast cancer and improved anti-cancer effects compared to monotherapy. The recent research conducted by Hu et al. (2021b) found that combined inhibition of PI3K/AKT/mTOR and MAPK/ERK signaling pathways has a better tumor suppression effect in GC. Our research has, for the first time, demonstrated that the upregulation of ASF1B can simultaneously activate both the PI3K/AKT and ERK1/2 signaling pathways, further underscoring the value of ASF1B as a potential therapeutic target in GC.

In conclusion, we have substantiated the upregulation of ASF1B expression in gastric cancer tissues through comprehensive bioinformatics analyses and experimental validation, affirming its function in facilitating tumor growth in both *in vivo* and *in vitro* models, as well as organoid systems. ASF1B was first found to promote gastric cancer progression by downregulating H2AC20 to affect the activation of PI3K/AKT and ERK1/2 signaling pathways. Overall, the cellular and animal models, along with the potential mechanisms explored in our research, present a departure from prior studies, deepening our insight into the role of ASF1B in gastric cancer and enhancing our comprehension of its involvement in the pathogenesis and advancement of this malignancy. Identified as an oncogene, ASF1B plays a significant role in tumor progression and holds considerable potential as a therapeutic target for gastric cancer. Despite its potential, targeting ASF1B therapeutically presents several challenges. One major challenge is the ubiquitous expression of ASF1B in various cancer types, which may complicate the development of specific inhibitors without affecting normal cells. Additionally, ASF1B’s involvement in multiple cellular processes, such as DNA replication and repair, could lead to unintended side effects if not precisely targeted. This study found that ASF1B and H2AC20 can interact with each other and regulate the expression of H2AC20. Based on this finding, we can try to design small molecule proteins that target the binding sites of the two to block the binding of the two, and explore the role of ASF1B as an anti-tumor target more safely and effectively.

## Data Availability

The raw data supporting the conclusions of this article will be made available by the authors, without undue reservation.

## Supplementary material

SUPPLEMENTARY FIGURE S1 Expression of ASF1B in the TISCH database **(A, B)**. CO-IP detection of ASF1B and H2AC20 in AGS cells **(C)**. Changes in H2AC20 mRNA levels in AGS cells after ASF1B knockdown **(D)**. WB detection of PI3K/AKT and ERK1/2 pathway proteins in ASF1B-overexpressing AGS cells **(E)**.
