# Supplementary figures and images for: ASF1B promotes gastric cancer progression by modulating H2AC20 and activating PI3K/AKT and ERK1/2 pathways
Source: Front Pharmacol. 2025 Feb 18;16:1533257. doi: 10.3389/fphar.2025.1533257 (PMC11876136; doi:10.3389/fphar.2025.1533257)

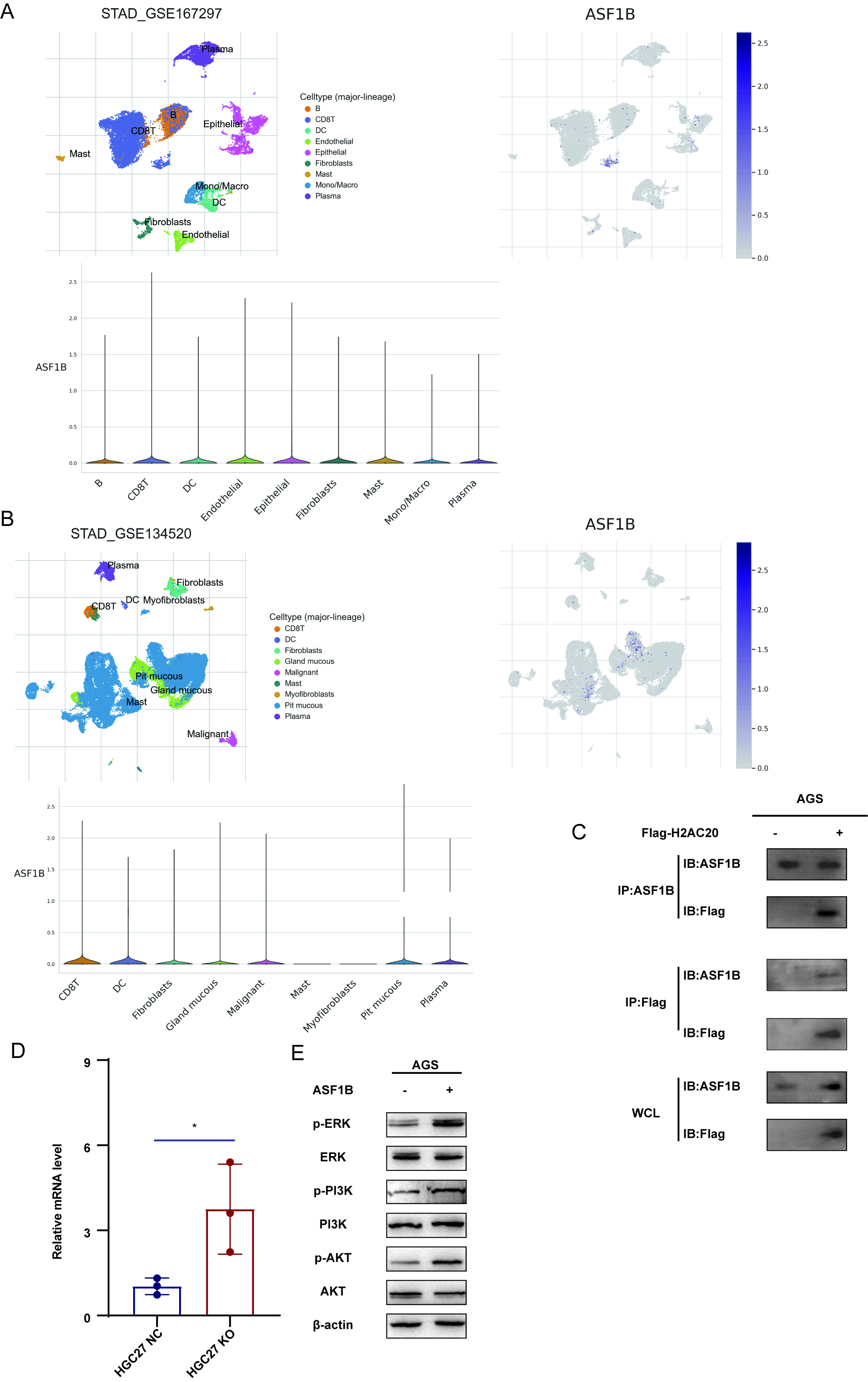

Supplement: Supplementary file 1 [file Image1.tif]
